# Effects of surgical masks on droplet dispersion under various oxygen delivery modalities

**DOI:** 10.1186/s13054-021-03512-w

**Published:** 2021-02-27

**Authors:** Takahiro Takazono, Kazuko Yamamoto, Ryuta Okamoto, Shimpei Morimoto, Koichi Izumikawa, Hiroshi Mukae

**Affiliations:** 1 Department of Respiratory Medicine, Nagasaki University Hospital, Nagasaki, Japan; 2 Department of Infectious Diseases, Nagasaki University Graduate School of Biomedical Sciences, 1-7-1 Sakamoto, Nagasaki, 852-8501 Japan; 3 Infection Control and Education Center, Nagasaki University Hospital, Nagasaki, Japan; 4 Visual Solution Division, Shin Nippon Air Technologies, Tokyo, Japan; 5 Clinical Research Center, Nagasaki University Hospital, Nagasaki, Japan; 6 Innovation Platform & Office for Precision Medicine, Nagasaki University Graduate School of Biomedical Sciences, Nagasaki, Japan

## Dear editor,

Aerosol dispersion under oxygen delivery modalities, including the high-flow nasal cannula (HFNC), is a critical concern for healthcare workers who have treated acute hypoxemic respiratory failure during the coronavirus disease (COVID-19) pandemic. Whether HFNC increases the aerosol dispersion is still controversial [1–3]. This study aimed to visualize and quantify dispersion particles under various oxygen delivery modalities and examine the protective effect of surgical masks on particle dispersion.

Three and five healthy men were voluntarily enrolled for video recording and quantification of particles, respectively. In the visualization experiment, three conditions, including room air, nasal canula at 5 L/min (non-humidified, Nakamura Medical Industry Co., Ltd.), and HFNC at 60 L/min (humidified, AIRVO2/Opti flow + , Fisher & Paykel Healthcare) were used. For quantitative evaluation, particle dispersion under four conditions including room air, nasal canula, and HFNC (30 or 60L/min) were tested. Particle dispersions during rest breathing for 30 s, speaking, and coughing were recorded three times each and automatically counted for five times each in the above conditions, and were evaluated with or without surgical masks. Dispersing droplets from mouths were flashed continuously by Parallel Eye D (Shin Nippon Air Technologies). Scattering light from droplets was recorded by a super high-sensitive camera (Eye Scope, Shin Nippon Air Technologies). The recording area was 1 m from participants' mouths. Particle dispersions were counted using the Fine Particle Visualization System (Type-S, Shin Nippon Air Technologies) with a 1/30 s speed, which was located in two linear columns at 25–45 and 60–80 cm from the mouth, respectively. Particles sized > 5 µm and > 0.5 µm were automatically counted independently. Differences in continuous numbers between the two groups were analyzed by ratio paired *t *test. A *p *value < 0.05 was considered statistically significant.

**Fig. 1:**
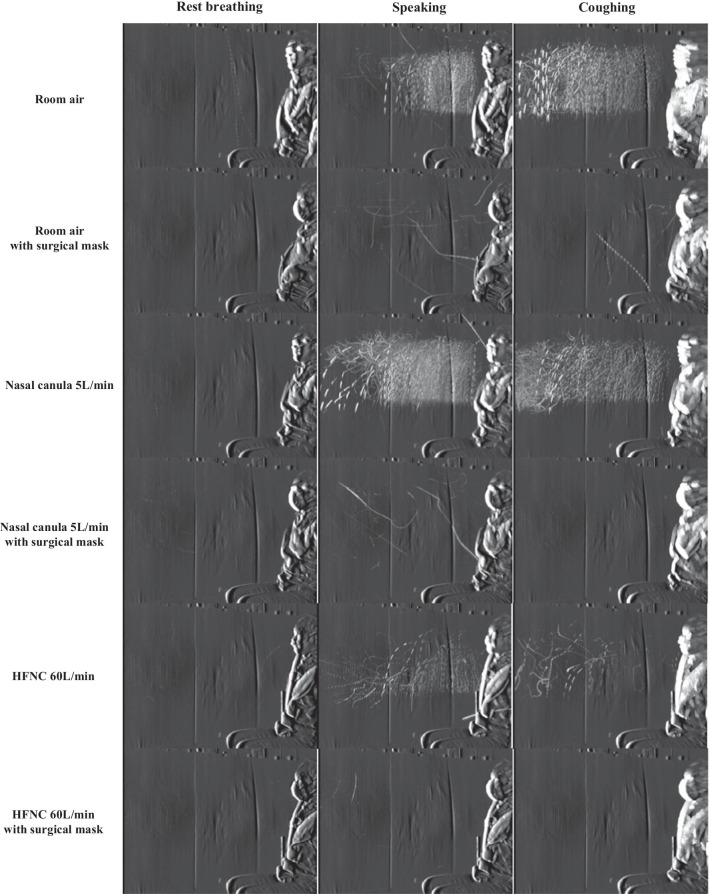
Representative accumulated photographs of droplet (> 5 µm) dispersion. Representative accumulated photographs at rest breathing, speaking, and coughing in room air, 5 L/min of nasal cannula, and 60 L/min of HFNC, and with or without surgical masks are shown. Droplet dispersion was not visually increased by oxygen delivery modalities compared to room air, regardless of breathing patterns. With surgical masks over the nasal canula or HFNC, droplet dispersion was barely visible. *HFNC* high-flow nasal cannula;

**Fig. 2:**
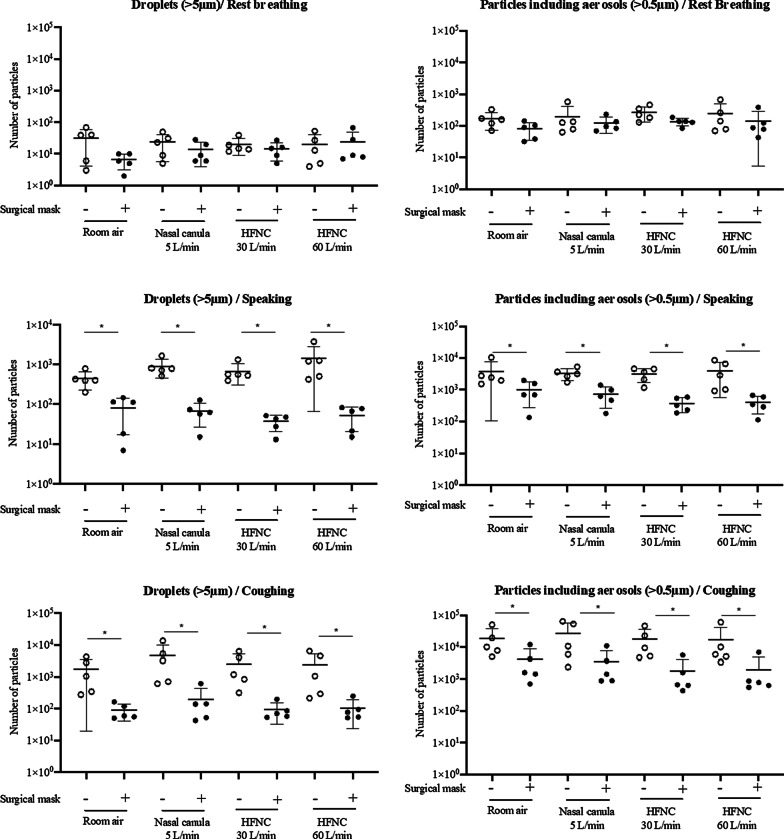
Number of droplets (> 5 µm) and particles including aerosols (> 0.5 µm). Number of droplets (> 5 µm) and particles including aerosols (> 0.5 µm) in room air under three different oxygen delivery modalities (5 L/min of nasal cannula, 30 L/min of HFNC, or 60 L/min of HFNC) and three breathing patterns (rest breathing, speaking, and coughing), with or without surgical masks, is shown. *HFNC* high-flow nasal cannula. *p < 0.05, ratio paired *t *test

The accumulated droplet (> 5 μm) dispersion in a representative participant is shown in Fig. 1, Coughing led to the maximum amount and distance of particle dispersion, regardless of modalities. Droplet dispersion was not visually increased by oxygen delivery modalities compared to room air, regardless of breathing patterns. With surgical masks over the nasal or high-flow nasal cannula, droplet dispersion was barely visible. Quantification results of particle dispersion are shown in Fig. 2. Particle dispersion counts at coughing showed a 1-log increase compared to those at speaking and more than a 2-log increase compared to those at rest breathing. Counts of droplets (> 5 μm) and smaller particles including aerosols (> 0.5 μm) were not different under nasal canula or HFNC compared to room air while speaking and coughing. Furthermore, the increased flow rate of HFNC (from 30 L/min to 60 L/min) did not affect the particle counts, even while coughing which was consistent with previous study [3]. Wearing surgical masks significantly decreased particle dispersion in all modalities while speaking and coughing; reduction rates were approximately 95% and 80–90% for droplets (> 5 μm) and smaller particles including aerosols (> 0.5 μm), respectively.

The main strength of this study is that particle dispersion imaging and counts under oxygen delivery modalities, recorded by highly sensitive instruments with controlled temperature and humidity, suggested that HFNC did not generate particles. Further, the effectiveness of surgical mask over HFNC was promising. However, our study was assessed in healthy volunteers, and therefore, it is not certain whether these results directly apply to the patients with viral pneumonia, as those patients might not be able to wear a mask appropriately.

In conclusion, HFNC did not increase droplet and aerosol dispersion compared to standard nasal cannula therapy, even at highest flow, and surgical masks over HFNC may be safely used for acute hypoxemic respiratory failure patients, including coronavirus disease patients.

## Data Availability

The datasets used and/or analyzed during the current study are available from the corresponding author on reasonable request.
